# Phytoplankton Composition and Their Related Factors in Five Different Lakes in China: Implications for Lake Management

**DOI:** 10.3390/ijerph19053135

**Published:** 2022-03-07

**Authors:** Junmei Jia, Qiuwen Chen, Haidong Ren, Renjie Lu, Hui He, Peiwen Gu

**Affiliations:** 1 State Key Laboratory of Plateau Ecology and Agriculture, Qinghai University, Xining 810016, China; mingqiangren@163.com (H.R.); hehui1113@sina.com (H.H.); gpw214206@163.com (P.G.); 2 State Key Laboratory of Hydrology-Water Resources and Hydraulic Engineering, Nanjing 210029, China; 3 CEER, Nanjing Hydraulic Research Institute, Nanjing 210029, China; 4 Key Laboratory of the Northern Qinghai-Tibet Plateau Geological Processes and Mineral Resources, Qinghai Geological Survey Institute, Xining 810012, China; 5 Jiangsu Suzhou Environmental Monitoring Center, Suzhou 215000, China; lurenjie990@163.com

**Keywords:** phytoplankton, environmental factors, connections, eutrophic lakes, alpine lakes

## Abstract

In this paper, two trophic lakes: Lake Taihu and Lake Yanghe, and three alpine lakes: Lake Qinghai, Lake Keluke, and Lake Tuosu, were investigated to discover the connections between environmental factors and the phytoplankton community in lakes with differences in trophic levels and climatic conditions. Three seasonal data, including water quality and phytoplankton, were collected from the five lakes. The results demonstrated clear differences in water parameters and phytoplankton compositions in different lakes. The phytoplankton was dominated by Bacillariophyta, followed by Cyanobacteria and Chlorophyta in Lake Qinghai, Lake Keluke, and Lake Tuosu. It was dominated by Cyanobacteria (followed by Chlorophyta and Bacillariophyta in Lake Yanghe) and Cyanobacteria (followed by Chlorophyta and Cryptophyta in Lake Taihu). The temperature was an essential factor favoring the growth of Cyanobacteria, Chlorophyta, and Bacillariophyta, especially Cyanobacteria and Chlorophyta. The pH had significantly negative relationships with Cyanobacteria, Chlorophyta, and Bacillariophyta. Particularly, a high pH might be a strong and negative factor for phytoplankton growth in alpine lakes. A high salinity was also an adverse factor for phytoplankton. Those results could provide fundamental information about the phytoplankton community and their correlated factors in the alpine lakes of the Tibetan Plateau, contributing to the protection and management of alpine lakes.

## 1. Introduction 

The phytoplankton community, as a crucial primary producer, has profound influences on the geochemical cycling and the function of aquatic ecosystems [1]. In many eutrophic aquatic systems around the world, the phytoplankton community is dominated by several bloom-forming species, and the blooming of phytoplankton threatens those aquatic systems. Previous studies have suggested that the phytoplankton, dominated by cyanobacteria, affect the zooplankton structure and weaken the zooplankton biodiversity [2]. Additionally, the toxic species have adverse effects on other aquatic organisms, and the toxins accumulate in their body [3]. Thus, the exploration of environmental factors influencing the structure and dynamic of phytoplankton in eutrophic lakes has caught the attention of scientists worldwide [4,5,6]. Lakes located in Tibetan Plateau, such as Lake Qinghai, are neglected since they have low phytoplankton abundance and present no phytoplankton blooms. However, endemic fish and rare birds live in those lakes, and the alteration of the phytoplankton may affect the survival of rare fish and birds. Climate change may lead to a severe situation in those alpine lakes, as they are more susceptible to climate change. Therefore, it is critical to reveal phytoplankton structures and their driving factors in both trophic lakes and alpine lakes. 

The factors affecting the growth of phytoplankton have been deeply explored in temperate and subtropical lakes. Temperature and nutrients are considered the most important factors regulating the growth of phytoplankton [1,7,8,9]. High temperature favors phytoplankton by increasing the growth rate and shifting the phytoplankton with higher optimum temperature species, such as cyanobacteria [10,11,12]. The effect of nutrients on cyanobacteria has been investigated in the long term. P has been identified as a limited nutrient factor in freshwater systems [13,14,15,16]. However, some buoyant species, including *Microcystis*, *Cylindrospermopsis*, *Anabaena*, *Aphanizomenon*, and *Gloeotrichia*, are not likely limited by P, because the buoyant species can vertically migrate, consume excess phosphorus at the sediment–water interface, and then rise to the water surface to form blooms [17,18,19,20,21]. N is also a critical factor for the growth of cyanobacteria. N loading may promote *Microcystis* blooms by not only the enhancement of growth [22,23,24,25] but also the synthesis of protease inhibitors, discouraging zooplankton grazing [26,27]. Additionally, the N_2_-fixing cyanobacteria, such as *Anabaena*, *Aphanizomenon*, *Aphanothece*, *Cylindrospermopsis*, and *Gloeotrichia*, exhibit great flexibility in the N sources to form blooms and N fixation, making N sufficient to allow biomass to be continuously produced [9,17,28,29]. Temperature and nutrients are also essential factors for phytoplankton growth in high-latitude lakes [30,31]. A nine-year study of a mountain lake in Austria demonstrated that long-term phytoplankton changes were mainly attributed to the increasing temperature, while nutrients acted as modulating factors regulating the short-term phytoplankton changes [30]. A study of Lake Qinghai suggested that the increase in P load under climate change and overgrazing favored the growth of P-limited phytoplankton [31]. Nevertheless, some environmental factors such as salinity may have been neglected in the previous study. Considering that phytoplankton blooms are seldom reported in brackish lakes, the bloom-forming cyanobacterium is restricted in saltwater. However, freshwater blooms are found in many coastal areas [32,33]. Thus, salinity may not be a decisive factor in bloom formation. It is urgent to explore the potential factors influencing the phytoplankton community in both fresh and brackish lakes under climate change. 

Lake Taihu and Lake Yanghe are eutrophic lakes located in subtropical and temperate areas, respectively. Lake Qinghai, Lake Keluke, and Lake Tuosu are alpine lakes located in the Tibetan Plateau. These five lakes have different climate conditions, presenting many differences in both environmental factors and the phytoplankton community. The comparisons between them could reveal the potential factors regulating the phytoplankton community. The exploration of the phytoplankton structure and the correlated factors is essential for understanding phytoplankton succession in different lakes, especially in the alpine lakes in the Tibetan Plateau. This study aimed to reveal the differences in phytoplankton communities of different lakes and explore the correlated factors regulating the growth of different phytoplankton phyla. 

## 2. Materials and Methods

### 2.1. Study Area and Sampling Locations

**Figure 1:**
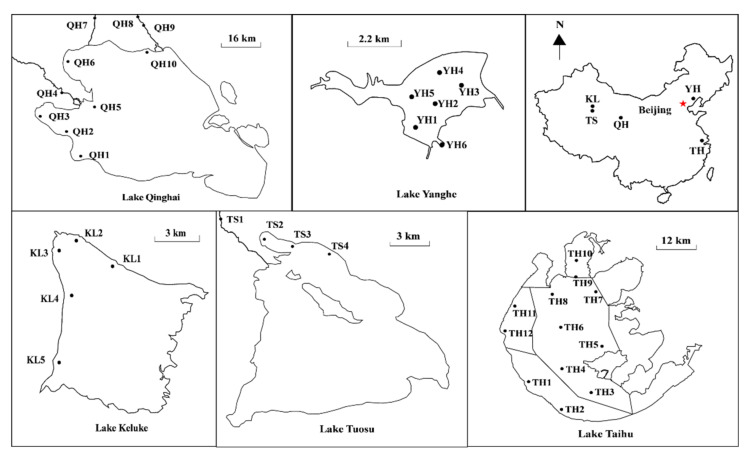
Sampling sites of Lake Qinghai, Lake Keluke, Lake Tuosu, Lake Taihu, and Lake Yanghe in China.

Lake Taihu is located at the center of the Yangtze River Delta in Eastern China (30°56′–31°33′ N, 119°56′–120°54′ E) (Figure 1), with an annual average air temperature of 15–17 °C. It has a surface area of approximately 2338 km^2^, a mean water depth of 1.9 m, and a maximum depth of 2.6 m [34]. Lake Taihu serves as a critical water resource for drinking, irrigation, aquaculture, and many industries, as well as recreation and tourism. Nonetheless, Lake Taihu has suffered from high nutrient loads and eutrophication in recent decades [35]. 

Lake Yanghe is located in Qinhuangdao City in Northern China (39°59′–40°12′ N, 119°10′–119°15′ E) (Figure 1), with an annual average air temperature of 11 °C. It has a drainage area of 755 km^2^, a mean water depth of 5.7 m, and a maximum depth of over 40 m (Li et al. 2020). Lake Yanghe is a hydraulic project on the Yanghe River, supplies industrial and domestic water to Qinhuangdao City, and provides flood control for Funing Country and Beidaihe downstream. In recent years, nutrients from surface runoffs have greatly increased due to industrial and economic development in the area, leading to the continued outbreak of cyanobacterial blooms in the summer. This has negatively influenced its function as a drinking water source. 

Lake Qinghai is located in Qinghai Province in Northwest China (36°32′–37°15′ N, 99°36′–100°47′ E) (Figure 1), with an area of about 4260 km^2^, a maximum depth of 30 m, and a mean surface elevation of 3194 m [36,37]. This area is characterized by an alpine and continental climate, with an annual average temperature of 1.2 °C [37]. Lake Keluke (37°15′–37°20′ N, 96°51′–96°58′) and Lake Tuosu (37°04′–37°13′ N, 96°50′–97°03′E) are located in Qinghai Province in Northwest China (Figure 1), with an annual average air temperature of 11 °C [38]. Lake Keluke has an area of about 56.7 km^2^, a mean water depth of 4 m, and a maximum depth of 13.3 m. Lake Tuosu has an area of about 145 km^2^ and a maximum depth of 25 m [38]. 

### 2.2. Sample Collection and Processing

Water samples of five different lakes were taken in the spring, summer, and autumn. Water samples of Lake Taihu and Lake Yanghe were collected in the spring, summer, and autumn of 2014. Lake Taihu samples were collected from 12 locations (Figure 1) on 16 April, 22 July, and 24 September 2014. Lake Yanghe samples were collected from 6 locations (Figure 1) on 15 May, 21 August, and 14 October 2014. Water samples of Lake Qinghai, Lake Keluke, and Lake Tuosu were collected in the spring, summer, and autumn of 2018. Water samples of Lake Qinghai were collected on 28 and 29 May, on 20 and 21 August, and on 23 and 24 October from 10 sites (Figure 1). Water samples of Lake Keluke were collected on 1 June, on 18 August, and on 22 October from 5 sites (Figure 1). Water samples of Lake Tuosu were collected on 2 June, on 19 August, and on 22 October from 4 sites (Figure 1). Surface water samples (2 L) were taken at a 0.5-m depth if the water depth was less than 2 m and were taken at 0.5 m, 1 m, 2 m, 3 m, and 4 m if the water depth was less than 5 m [39]. All the samples were stored in polyethylene barrels at 4 °C in the dark before laboratory analysis. Water temperature, pH, and salinity were measured in situ at each site using a YSI 6600 multi-probe sonde (YSI, Yellow Springs, OH, USA). An appropriate amount of water samples was filtered through GF/C glass fiber filters (Whatman, Kent, UK). Meanwhile, total nitrogen (TN) and total phosphorus (TP) were measured using the Chinese standard methods of HJ/T 636-2012 and GB/T 11983-1989, respectively. 

Water samples (100 mL) taken from each sampling site were treated with Lugol’s iodine solution to fix phytoplankton species. Phytoplankton was settled in 20 mm × 20 mm chambers and identified/enumerated by light microscopy following commonly used monographs on phytoplankton [40,41]. The Shannon–Wiener index (*H*) based on the number was calculated for phytoplankton [42]. The biodiversity of phytoplankton in the five lakes was calculated according to Bacillariophyta, Cyanobacteria, Chlorophyta, Cryptophyta, Pyrrophyta, Euglenophyta, and Chrysophyta.

### 2.3. Statistical Analysis

All data were statistically processed using SPSS 17.0 (SPSS, Inc., Chicago, IL, USA, 2008). The frequency of the water parameters and phytoplankton abundance approximated normal distributions. Pearson correlations were adopted to assess the relationships between environmental factors and phytoplankton. Additionally, the regression models were fitted to the water parameters and phytoplankton with significant relationships. 

## 3. Results

### 3.1. Water Parameters and Nutrients in the Five Lakes

Table 1 exhibited the seasonal changes of the water parameters and nutrient levels. Lake Taihu had the highest average temperature of the three seasons, followed by Lake Yanghe, Lake Tuosu, and Lake Keluke, and lowest in Lake Qinghai (*p* < 0.05). The autumn had lower water temperatures than in the spring in the northern lakes, including Lake Qinghai, Lake Keluke, Lake Tuosu, and Lake Yanghe, with a higher temperature than that in the spring for Lake Taihu. Lake Qinghai and Lake Tuosu had the highest pH in the five lakes (*p* < 0.05), followed by Lake Keluke and Lake Taihu (*p* < 0.05). Lake Yanghe had the lowest pH in the five lakes (*p* < 0.05). There were significant differences in the salinity in the five lakes. Lake Tuosu had the highest salinity in the five lakes, followed by Lake Qinghai and Lake Keluke. Lake Taihu and Lake Yanghe had the lowest salinity in the five lakes. The inflow rivers had a lower temperature, pH, and salinity compared to Lake Qinghai. This is also true in Lake Tuosu. 

There was a clear seasonal pattern in TN in Lake Yanghe and Lake Taihu. The mean TN in Lake Yanghe were 2.7 ± 0.02 mg/L, 0.1 ± 0.01 mg/L, and 0.5 ± 0.1 mg/L in the spring, summer, and autumn, respectively. The mean TN in Lake Taihu were 2.8 ± 0.7 mg/L, 0.6 ± 0.5 mg/L, and 0.5 ± 0.4 mg/L in the spring, summer, and autumn, respectively. The TP concentrations of the five lakes were all lower than 0.1 mg/L. Lake Taihu, Lake Qinghai, and Lake Keluke had higher TP compared to Lake Yanghe and Lake Tuosu. There were not many differences in TP concentrations of Lake Keluke and Lake Tuosu in different seasons. There were clear seasonal differences in Lake Qinghai, Lake Taihu, and Lake Yanghe. The TN was higher in the inflow rivers than the main lakes of both Lake Qinghai and Lake Tuosu, while it had an opposite pattern about TP in the inflow rivers and the main lakes in the two lakes. 

### 3.2. Phytoplankton Composition and Biodiversity in the Five Lakes

**Figure 2:**
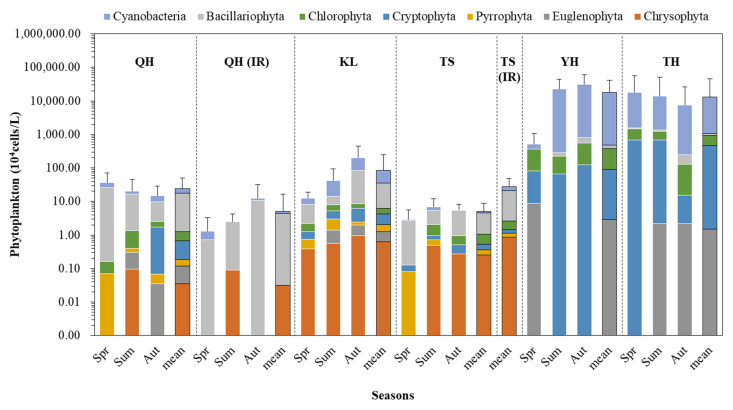
Phytoplankton composition based on the abundance in different seasons in Lake Qinghai (QH), Lake Keluke (KL), Lake Tuosu (TS), Lake Yanghe (YH), Lake Taihu (TH), and the inflow rivers (IR) of Lake Qinghai and Lake Tuosu. The error bars indicate the standard deviations of phytoplankton abundance in each lake.

The composition of phytoplankton differed among the five lakes (Figure 2). The dominant phylum in Lake Qinghai, Lake Keluke, and Lake Tuosu was Bacillariophyta in the spring, summer, and autumn. In Lake Qinghai, the phytoplankton abundance was 3.7 × 10^5^ cells/L, 2.0 × 10^5^ cells/L, and 1.5 × 10^5^ cells/L. In the spring, summer, and autumn, Bacillariophyta accounted for 65%, 59%, and 39%, and Cyanobacteria accounted for 21%, 9%, and 37%, respectively. In Lake Keluke, it was 1.2 × 10^5^ cells/L, 4.2 × 10^5^ cells/L, and 2.0 × 10^7^ cells/L. Bacillariophyta accounted for 56%, 40%, and 50% of phytoplankton, and Cyanobacteria accounted for 24%, 31%, and 28% in the spring, summer, and autumn, respectively. In Lake Tuosu, it was 2.9 × 10^4^ cells/L, 6.9 × 10^4^ cells/L, and 5.6 × 10^4^ cells/L. Bacillariophyta accounted for 88%, 47%, and 82% of phytoplankton, and Cyanobacteria accounted for 9%, 22%, and 4% in the spring, summer, and autumn, respectively. The phytoplankton abundance of Lake Yanghe was 5.2 × 10^6^ cells/L, 2.3 × 10^8^ cells/L, and 3.1 × 10^8^ cells/L in the spring, summer, and autumn, respectively. The dominant phylum of Lake Yanghe was Cyanobacteria in all three seasons, except for the spring. Chlorophyta accounted for 52% of phytoplankton in Lake Yanghe in the spring, and Cyanobacteria accounted for 89% and 98% of phytoplankton in Lake Yanghe in the summer and autumn, respectively. The dominant phylum of Lake Taihu was Cyanobacteria, which accounted for 35%, 44%, and 43% of phytoplankton in the spring (3.4 × 10^8^ cells/L), summer (2.6 × 10^8^ cells/L), and autumn (1.5 × 10^8^ cells/L), respectively. The dominant species was *Microcystis* sp. in Lakes Taihu and Yanghe and was *Synedra* sp. In Lake Qinghai, Lake Keluke, and Lake Tuosu. The Bacillariophyta species (*Navicula* sp. And *Cyclotella* sp.); the Chlorophyta species (*Monoraphidium* sp., *Schroederia* sp., *Oocystis* sp., *Scenedesmus* sp., *Chlamydomonas* sp., and *Closterium* sp.); and the Cyanobacteria species (*Anabeana* sp. and *Oscillatoria* sp.) were discovered in all five lakes. 

There were clear differences in phytoplankton biodiversity of the five lakes. In Lake Qinghai, the phytoplankton biodiversity was 0.19 ± 0.30, 0.38 ± 0.37, and 0.39 ± 0.46 in the spring, summer, and autumn, respectively. In Lake Keluke, the phytoplankton biodiversity was 0.87 ± 0.56, 0.75 ± 0.67, and 0.72 ± 0.45, respectively. In Lake Tuosu, the phytoplankton biodiversity was 0.41 ± 0.39, 1.25 ± 0.19, and 0.66 ± 0.30 in the spring, summer, and autumn, respectively. In Lake Yanghe, the phytoplankton biodiversity was 0.91 ± 0.18, 0.10 ± 0.07, and 0.34 ± 0.43 in the spring, summer, and autumn, respectively. In Lake Taihu, the phytoplankton biodiversity was 0.38 ± 0.33, 0.51 ± 0.37, and 0.41 ± 0.39, respectively. 

### 3.3. Correlations between Water Parameters and Phytoplankton in the Five Lakes

**Figure 3:**
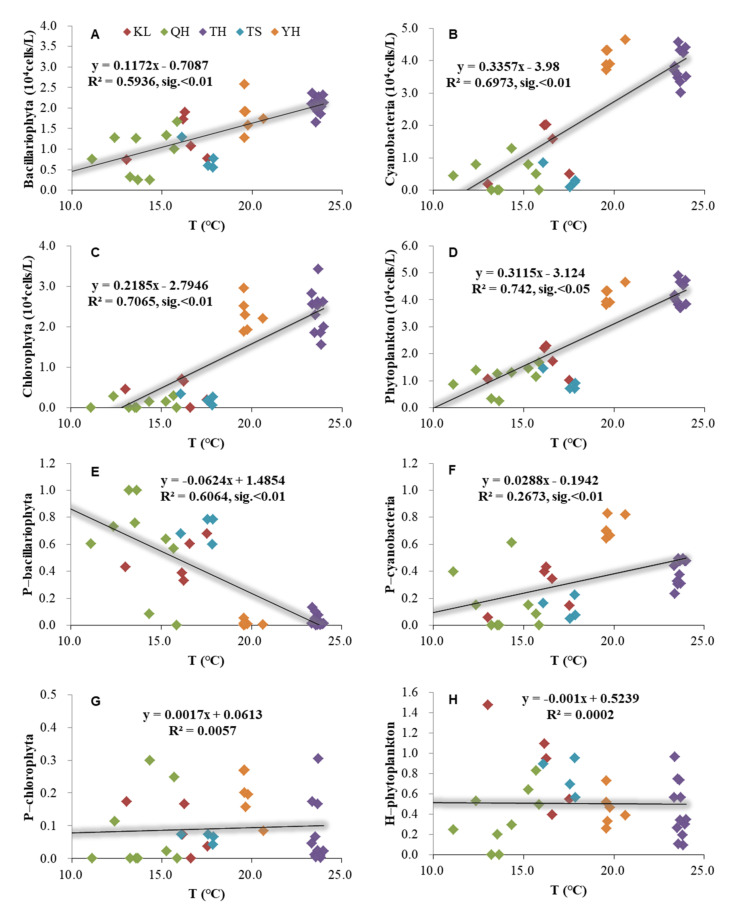
Relationships between temperature (T) and phytoplankton based on the annual average data of each site of Lake Qinghai, Lake Keluke, Lake Tuosu, Lake Yanghe, and Lake Taihu. (**A**–**D**) showed the relationships between T and phytoplankton, and (**E**–**H**) showed the relationships between T and phytoplankton composition. Bacillariophyta, Chlorophyta, Cyanobacteria, and phytoplankton abundance were transformed by Log (X + 1). P-i indicates the i/phytoplankton ratio based on abundance. H-phytoplankton represents the biodiversity of phytoplankton.

The phytoplankton had significant relationships with temperature based on the annual average data from Lake Qinghai, Lake Keluke, Lake Tuosu, Lake Yanghe, and Lake Taihu (Figure 3). Bacillariophyta, Cyanobacteria, Chlorophyta, and phytoplankton abundance had significantly positive relationships with temperature under R^2^ = 0.59, 0.70, 0.71, and 0.74 (*p* < 0.05). Additionally, the ratio of Bacillariophyta/phytoplankton had a significantly negative relationship with temperature (*p* < 0.01), and the ratio of Cyanobacteria/phytoplankton had a significantly positive relationship with temperature (*p* < 0.01). 

**Figure 4:**
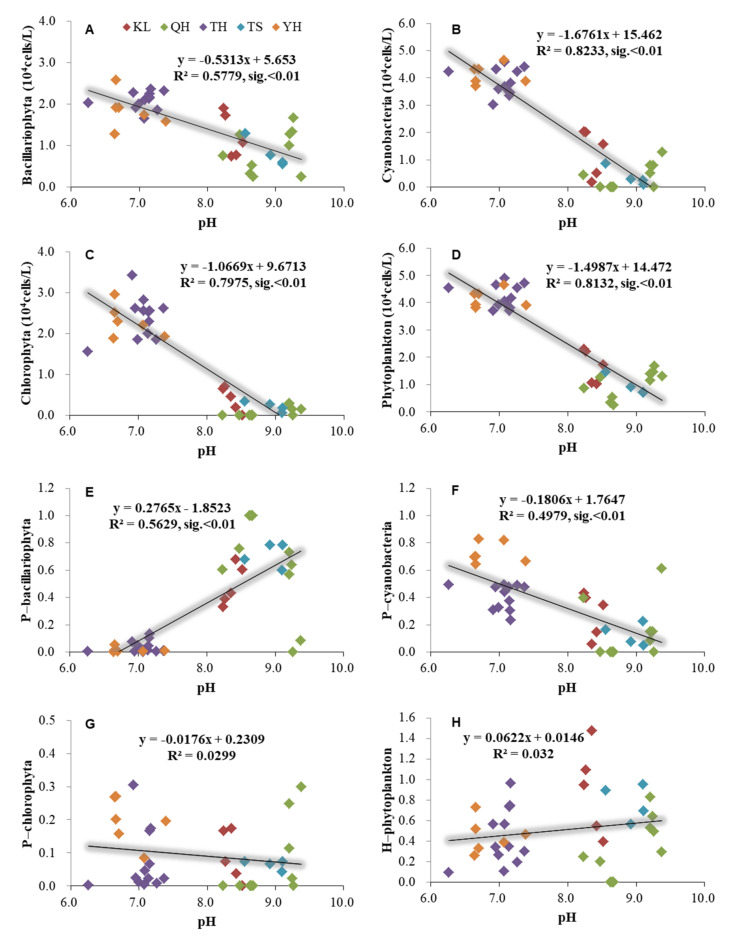
Relationships between pH and phytoplankton based on the annual average data of each site of Lake Qinghai, Lake Keluke, Lake Tuosu, Lake Yanghe, and Lake Taihu. (**A**–**D**) showed the relationships between pH and phytoplankton, and (**E**–**H**) showed the relationships between pH and phytoplankton composition. Bacillariophyta, Chlorophyta, Cyanobacteria, and phytoplankton abundance were transformed by Log (X + 1). P-i indicates the i/phytoplankton ratio based on abundance. H-phytoplankton represents the biodiversity of phytoplankton.

**Figure 5:**
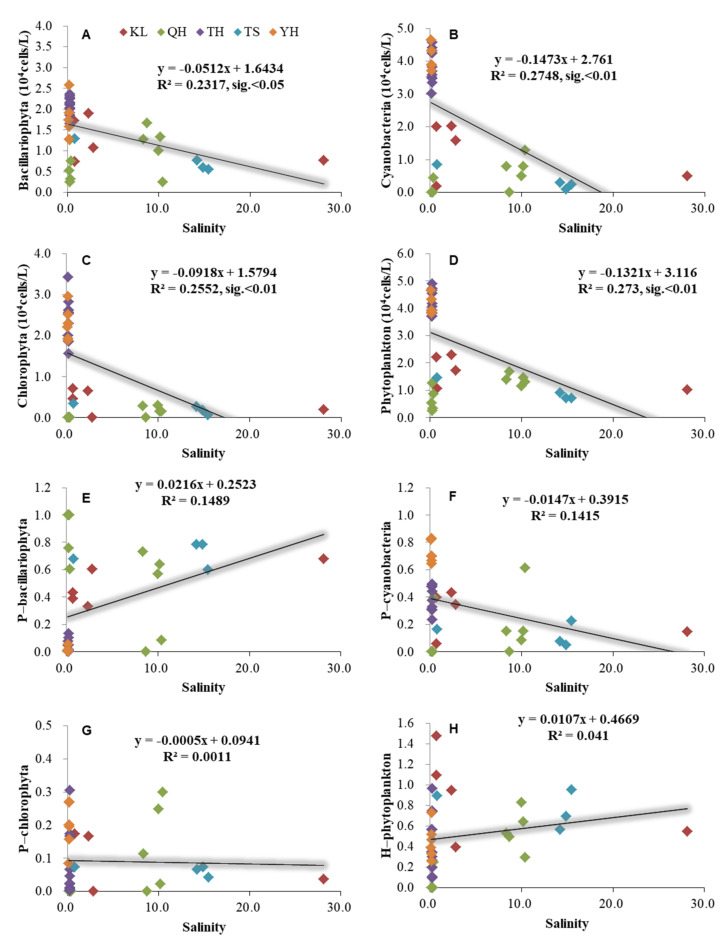
Relationships between salinity and phytoplankton based on the annual average data of each site of Lake Qinghai, Lake Keluke, Lake Tuosu, Lake Yanghe, and Lake Taihu. (**A**–**D**) showed the relationships between salinity and phytoplankton, and (**E**–**H**) showed the relationships between salinity and phytoplankton composition. Bacillariophyta, Chlorophyta, Cyanobacteria, and phytoplankton abundance were transformed by Log (X + 1). P-i indicates the i/phytoplankton ratio based on abundance. H-phytoplankton represents the biodiversity of phytoplankton.

The phytoplankton had a significant relationship with pH based on the annual average data from the five lakes in China (Figure 4). Bacillariophyta, Cyanobacteria, Chlorophyta, and phytoplankton abundance had significantly negative relationships with pH under R^2^ = 0.58, 0.82, 0.80, and 0.81 (*p* < 0.01). Additionally, the ratio of Bacillariophyta/phytoplankton had a significantly positive relationship with the pH (*p* < 0.01). The ratio of Cyanobacteria/phytoplankton had a significantly negative relationship with the pH (*p* < 0.01). Additionally, phytoplankton had a significant relationship with salinity based on the annual average data from the five lakes in China (Figure 5). Bacillariophyta, Cyanobacteria, Chlorophyta, and phytoplankton abundance had significant negative relationships with salinity (*p* < 0.05) (Figure 5). 

## 4. Discussion

The temperature was one of the most essential factors regulating the growth of phytoplankton [1,7]. In the present study, phytoplankton abundance had a significantly positive relationship with water temperature. Additionally, the lakes with higher temperatures generally possessed higher phytoplankton abundance, such as Lake Taihu and Lake Yanghe. Thus, the temperature was a crucial factor promoting the growth of phytoplankton in the five lakes. Previous studies demonstrated that higher temperatures favored the growth of Cyanobacteria [10,11,12]. This is consistent with the result in the present study that the lakes with higher temperatures generally had higher Cyanobacteria abundance and a Cyanobacteria/phytoplankton ratio. Lake Taihu and Lake Yanghe had higher average water temperatures compared to Lake Qinghai, Lake Keluke, and Lake Tuosu. Hence, Lake Taihu and Lake Yanghe had higher average Cyanobacteria abundance and a higher Cyanobacteria/phytoplankton ratio than Lake Qinghai, Lake Keluke, and Lake Tuosu. Additionally, the Cyanobacteria/phytoplankton ratio exhibited a significantly positive relationship with temperature. 

The nutrients were another vital factor regulating the growth of phytoplankton. In the present study, TN had a high concentration in the five lakes, and there were not many differences in TP concentration. A previous study revealed that the growth of the dominant phytoplankton was not nutrient-limited under P enrichment ≥ 0.20 mg∙L^−1^ (P) and N enrichment ≥ 0.80 mg∙L^−1^ (N) [8]. Therefore, the growth of the dominant phytoplankton in all the lakes was not N-limited. In other words, TN does not shape the differences in phytoplankton abundance in the five lakes. Phosphorus is one of the limiting factors for phytoplankton growth in all five lakes, since the TP was much lower than 0.20 mg∙L^−1^ in those lakes. Thus, TP does not shape the differences in phytoplankton abundance in the five lakes. However, the high nutrient level in Lake Qinghai, Lake Keluke, and Lake Tuosu, located in the Tibetan Plateau, should be stressed under climate change. In recent years, the lakes in the Tibetan Plateau are undergoing an increase in temperature and precipitation. The rising temperature may result in a blooming of phytoplankton, especially Cyanobacteria, in those lakes with enough nutrients. The increase in precipitation may induce more nutrient input, especially TP, in the lakes of the Tibetan Plateau. This has been confirmed in Lake Qinghai that the TP increased but TN decreased in the inflowing rivers, contributing to the alleviation of the P deficiency in Lake Qinghai and the promotion of the growth of phytoplankton [31]. 

In the present study, the pH had significantly negative relationships with Bacillariophyta, Cyanobacteria, Chlorophyta, and phytoplankton. This may be caused by a relatively high phytoplankton abundance and low pH in Lake Taihu and Lake Yanghe, as well as relatively low phytoplankton abundance and high pH in other lakes. A previous study suggested that the natural phytoplankton biomass decreased in high pH (9.5) incubation, while the phytoplankton biomass increased in pH 8–9 incubation [43]. In Lake Taihu and Lake Yanghe, the mean pH was low (pH = 7.0 and 6.9, respectively), and the phytoplankton could grow well if the pH increased from 7.0 to 9.0, since the blooming of the phytoplankton was associated with an increase in pH [44]. This would be explained by the blooming of the phytoplankton, which depletes the dissolved CO_2_ concentration and, therefore, results in an increase in pH [45,46,47]. This is consistent with previous studies that the pH increased with increasing the cell densities of Cyanobacteria, and the Cyanobacteria blooms were associated with high pH [44,48,49]. In Lake Yanghe, the Cyanobacteria accounted for 98% of the total phytoplankton when the Cyanobacteria abundance reached 2.2 × 10^8^ cells/L, and the pH reached 8.7 in the summer. However, the high pH may not favor the growth of phytoplankton. Additionally, *Microcystis aeruginosa* and *Scenedesmus quadricauda* were at the stationary phase under high pH values (10.0), and both algae resumed growing when the pH was decreased using HCl [50]. Additionally, Touloupakis et al. [51] confirmed that the light conversion efficiency of Cyanobacteria decreased linearly with the increase in pH at a range of 7.5–11. Therefore, a high pH (high OH^−^) could cause an adverse effect on the phytoplankton growth [52], and the phytoplankton cannot grow well in Lake Qinghai and Lake Tuosu at high pH values (both mean pH ≥ 9.0). 

High salinity was an adverse factor for phytoplankton growth, since it can cause oxidative stress to algal cells, resulting in cell death [53,54]. This is consistent with the results in the present study. The Bacillariophyta, Cyanobacteria, Chlorophyta, and phytoplankton all had significantly negative relationships with salinity. However, the low phytoplankton abundance in some brackish lakes should not be simply attributed to high salinity. Recently, more and more studies have revealed that freshwater phytoplankton blooms occur in brackish waters [32,33]. Additionally, indoor cultivation has implied that the freshwater strain *Microcystis aeruginosa*, a typical blooming species, acclimated to a salinity gradient that could reach 7.5 [52]. In the present study, Lake Keluke had a salinity lower than the maximum tolerance salinity based on indoor cultivation but a low phytoplankton abundance. Hence, the low phytoplankton abundance of Lake Keluke should not be simply attributed to high salinity. In Lake Tuosu, nevertheless, the salinity reached 11.3 ± 9.2 and could have an adverse effect on many phytoplankton species. 

The factors elaborated above are imperative to phytoplankton, especially for phytoplankton of the alpine lakes in the Tibetan Plateau. The lakes in the Tibetan Plateau experience a warm and wet climate [55,56], leading to changes in the water temperature, nutrients, and salinity and eventually facilitating the growth of phytoplankton [57]. Particularly, phytoplankton blooms may occur in those brackish lakes with enough nutrients under climate change. The brackish lakes in the Tibetan Plateau should receive priority for management, since some rare fish and birds live in those lakes. Their simple and fragile food chains could be altered under climate change. Buffer zones may be an effective approach for those alpine lakes to alleviate diffusive pollutions from agriculture and livestock. 

## 5. Conclusions

The phytoplankton community was dominated by Bacillariophyta, followed by Cyanobacteria and Chlorophyta in the alpine lakes, including Lake Qinghai, Lake Keluke, and Lake Tuosu. It was dominated by Cyanobacteria, followed by Chlorophyta and Bacillariophyta in Lake Yanghe. Additionally, it was dominated by Cyanobacteria, followed by Chlorophyta and Cryptophyta in Lake Taihu. The temperature was a crucial factor influencing the growth of Cyanobacteria, Chlorophyta, and Bacillariophyta, especially Cyanobacteria and Chlorophyta. The pH had significantly negative relationships with Cyanobacteria, Chlorophyta, and Bacillariophyta. Moreover, it could be a strongly negative factor for phytoplankton growth in alpine lakes. Salinity had significantly negative relationships with Cyanobacteria, Chlorophyta, and Bacillariophyta. It was also an adverse factor for phytoplankton when it was very high. 

## Figures and Tables

**Table 1:** Means and standard deviations of several water parameters of lakes and inflow rivers of five lakes in China.

Lakes and Rivers (n)	Seasons	Water Parameters				
		Temperature (°C)	pH	Salinity (ppt)	TN (mg/L)	TP (mg/L)
Qinghai (6)	Spring	17.9 ± 2.3	9.2 ± 0.09	10.5 ± 0.7	2.7 ± 1.2	0.06 ± 0.05
	Summer	17.5 ± 0.1	9.2 ± 0.01	9.5 ± 1.1	1.8 ± 0.8	0.01 ± 0.004
	Autumn	7.0 ± 3.5	9.4 ± 0.2	8.6 ± 2.9	3.5 ± 3.0	0.04 ± 0.04
	**Mean**	**14.6 ± 5.5**	**9.3 ± 0.1**	**9.6 ± 1.8**	**2.6 ± 1.8**	**0.04 ± 0.04**
Qinghai (IR) (4)	Spring	12.3 ± 3.4	8.5 ± 0.3	0.26 ± 0.1	7.0 ± 9.6	0.004 ± 0.003
	Summer	16.5 ± 0.8	8.6 ± 0.3	0.28 ± 0.07	5.8 ± 4.9	0.02 ± 0.02
	Autumn	8.1 ± 0.9	8.6 ± 0.1	0.24 ± 0.09	7.0 ± 3.6	0.02 ± 0.01
	**Mean**	**12.5 ± 4.0**	**8.6 ± 0.2**	**0.26 ± 0.09**	**6.6 ± 6.6**	**0.01 ± 0.01**
Keluke (5)	Spring	20.6 ± 4.0	8.5 ± 0.2	10.2 ± 20.4	5.1 ± 8.0	0.05 ± 0.05
	Summer	21.2 ± 0.8	8.4 ± 0.3	3.0 ± 3.8	3.9 ± 1.1	0.05 ± 0.03
	Autumn	6.0 ± 0.6	8.2 ± 0.4	7.7 ± 11.7	7.5 ± 7.3	0.06 ± 0.05
	**Mean**	**15.9 ± 7.6**	**8.4 ± 0.3**	**6.9 ± 13.1**	**5.5 ± 6.0**	**0.05 ± 0.04**
Tuosu (3)	Spring	20.3 ± 0.9	9.2 ± 0.03	22.2 ± 0.4	0.3 ± 0.4	0.02 ± 0.002
	Summer	23.1 ± 1.2	8.8 ± 0.3	8.5 ± 7.4	2.0 ± 0.4	0.02 ± 0.003
	Autumn	10.1 ± 0.3	9.1 ± 0.02	13.9 ± 6.7	1.6 ± 0.3	0.02 ± 0.01
	**Mean**	**17.8 ± 5.9**	**9.0 ± 0.2**	**14.9 ± 7.8**	**1.3 ± 0.8**	**0.02 ± 0.008**
Tuosu (IR)(1)	**Mean**	**16.1 ± 6.8**	**8.6 ± 0.2**	**0.77 ± 0.1**	**2.2 ± 0.05**	**0.01 ± 0.004**
Yanghe (6)	Spring	17.1 ± 0.7	5.7 ± 0.8	0.17 ± 0.08	2.7 ± 0.02	0.01 ± 0.001
	Summer	26.9 ± 0.7	8.4 ± 0.2	0.16 ± 0.005	0.1 ± 0.01	0.01 ± 0.002
	Autumn	15.5 ± 0.1	6.5 ± 0.6	0.18 ± 0.001	0.5 ± 0.1	0.03 ± 0.01
	**Mean**	**19.8 ± 5.2**	**6.9 ± 1.3**	**0.17 ± 0.04**	**1.1 ± 1.2**	**0.02 ± 0.01**
Taihu (12)	Spring	17.4 ± 0.2	6.8 ± 0.3	0.30 ± 0.04	2.8 ± 0.7	0.09 ± 0.11
	Summer	30.6 ± 0.4	7.4 ± 0.4	0.24 ± 0.03	0.6 ± 0.5	0.01 ± 0.003
	Autumn	23 ± 0.4	6.9 ± 0.3	0.22 ± 0.02	0.5 ± 0.4	0.04 ± 0.03
	**Mean**	**23.7 ± 5.5**	**7.0 ± 0.4**	**0.25 ± 0.04**	**1.3 ± 1.2**	**0.05 ± 0.07**

n indicates the number of sampling sites; IR indicates inflow rivers.

## Data Availability

The data presented in this study are available on request from the corresponding author. The data are not publicly available due to privacy.
